# Emotional Experience and Awareness of Self: Functional MRI Studies of Depersonalization Disorder

**DOI:** 10.3389/fpsyg.2016.00432

**Published:** 2016-06-02

**Authors:** Nick Medford, Mauricio Sierra, Argyris Stringaris, Vincent Giampietro, Michael J. Brammer, Anthony S. David

**Affiliations:** ^1^Department of Psychiatry, Brighton and Sussex Medical SchoolBrighton, UK; ^2^Sackler Centre for Consciousness Science, University of SussexBrighton, UK; ^3^Institute of Psychiatry, Psychology, and NeuroscienceLondon, UK

**Keywords:** depersonalization, insula, interoception, self-awareness, fMRI

## Abstract

This paper presents functional MRI work on emotional processing in depersonalization disorder (DPD). This relatively neglected disorder is hallmarked by a disturbing change in the quality of first-person experience, almost invariably encompassing a diminished sense of self and an alteration in emotional experience such that the sufferer feels less emotionally reactive, with emotions experienced as decreased or “damped down,” so that emotional life seems to lack spontaneity and subjective validity. Here we explored responses to emotive visual stimuli to examine the functional neuroanatomy of emotional processing in DPD before and after pharmacological treatment. We also employed concurrent skin conductance measurement as an index of autonomic arousal. In common with previous studies we demonstrated that in DPD, there is attenuated psychophysiological response to emotional material, reflected in altered patterns of (i) regional brain response, (ii) autonomic responses. By scanning participants before and after treatment we were able to build on previous findings by examining the changes in functional MRI response in patients whose symptoms had improved at time 2. The attenuation of emotional experience was associated with reduced activity of the insula, whereas clinical improvement in DPD symptoms was associated with increased insula activity. The insula is known to be implicated in interoceptive awareness and the generation of feeling states. In addition an area of right ventrolateral prefrontal cortex emerged as particularly implicated in what may be “top-down” inhibition of emotional responses. The relevance of these findings to the wider study of emotion, self-related processes, and interoception is discussed.

## Introduction

Depersonalization disorder (DPD) is characterized by a persistent and distressing alteration in the quality of subjective experience, such that the individual experiences both themselves (depersonalization) and their surroundings (derealization) as oddly estranged and unreal. Although depersonalization and derealization are separable phenomena, in practice they usually co-occur (Sierra and David, 2011). In recent years there has been a growing appreciation that the phenomenology of DPD entails a sense of alienation and estrangement from experience in general, and in keeping with this, DPD patients often describe a reduced capacity for emotional response and a generalized reduction in the experience of bodily sensation. In phenomenological literature, the terms “de-affectualization” and “de-somatization” have been applied to these aspects of the condition. Two large factor analyses (Sierra et al., 2005; Simeon et al., 2008) of DPD symptomatology have confirmed emotional numbing (i.e., “de-affectualization”) as a key symptom domain in DPD. There are sound theoretical reasons for believing that this alteration in emotional experience is central to the condition (Medford, 2012), and it is very rare to encounter a patient who fulfills criteria for a diagnosis of DPD but does not describe this deficit of subjective emotional response.

A number of previous fMRI and psychophysiological studies of DPD have focused specifically on this emotional aspect of the condition, showing that autonomic response to emotionally salient visual stimuli is significantly reduced in DPD (Sierra et al., 2002), and that the changes in regional brain activity seen in response to emotional stimuli in healthy controls are largely absent in DPD (Phillips et al., 2001; Medford et al., 2006). These findings can be seen as plausible biological correlates of the subjective deficit of emotional experience described by patients with DPD (Medford, 2012).

In this study, patients with primary DPD were studied using an emotional vs. neutral block-design fMRI paradigm employed in an earlier imaging study of DPD (Phillips et al., 2001), utilizing stimuli drawn from the International Affective Picture System (IAPS, Bradley and Lang, 1999). The IAPS is a library of images that have been given normative ratings for emotional salience and arousal and which are widely used in emotion research. Patients were scanned before and after receiving pharmacological treatment (lamotrigine) specifically tailored to DPD (Sierra et al., 2001, 2006). Thus patients were scanned twice at two different time points (14 patients scanned at time 1, of whom 10 were re-scanned at time 2). The idea of this approach is twofold: scanning at time 1 represents an attempt to replicate previous findings, in a group of as yet untreated (i.e., medication-free) patients with DPD. Given the paucity of literature on this condition, it is important to attempt this kind of replication in order to explore the robustness of previous findings. Secondly, re-scanning at time 2 allows an assessment of the effects of pharmacological treatment on the neural response to emotional stimuli in this patient group, and how this relates to changes in clinical state.

Specific hypotheses were as follows:

1. That functional MRI results in DPD patients at time 1 would essentially replicate previous findings using IAPS images in a DPD patient group (Phillips et al., 2001) in showing a reduced response to emotionally salient stimuli, and a relative lack of difference in the response to emotional material compared to neutral. More specifically, it was predicted that right ventrolateral prefrontal cortex (Brodmann area 47) would be significantly activated in the emotional phase in DPD patients at time 1, but not in the controls, as in the Phillips et al. study. It was also predicted that healthy controls would show a response to emotional images characterized by significant activation in the emotional phase in areas known to be involved in emotional processing, specifically anterior insula, and sensory (in this case visual) cortex activation related to modulation of sensory cortex by affective processing.
2. That at time 2, DPD patients reporting a significant reduction in their symptoms would show a pattern of neural responses to emotional stimuli more akin to that seen in healthy controls, whereas those reporting no such reduction would continue to show a response pattern similar to that seen at time 1. In particular, because of prior work implicating underactivity of the anterior insula in DPD (discussed in detail below), we hypothesized that DPD patients who had improved clinically would at time 2 show significantly increased activation, compared to time 1, of anterior insula in response to emotional material.
3. That DPD patients would show generally reduced skin conductance responses (SCRs) to IAPS stimuli compared to healthy controls, and that at time 2 this effect would be influenced by response to treatment in a similar manner to the fMRI findings i.e., that DPD patients who improved clinically would, at time 2, have an SCR profile resembling that seen in controls, with significantly more autonomic response to emotional stimuli than in the same patients at time 1 or non-improved patients at time 2.

The key hypothesis, in its simplest form, was that patients who show a significant response to treatment [defined as a 30% or greater reduction in Cambridge Depersonalization State Scale (CDSS) scores at time 2 relative to time 1 (Sierra et al., 2001, 2006), accompanied by subjective reported improvement at clinical follow-up] would also show a concomitant change in their neural and physiological response patterns.

## Participants and methods

Fourteen adult patients were recruited from the Depersonalization Clinic at the Maudsley Hospital. Every patient had a diagnostic assessment performed by a psychiatrist experienced in the evaluation and treatment of DPD (either NM or MS). Patients were recruited according to the following inclusion and exclusion criteria: (1) Meeting diagnostic criteria for primary DPD. Where other psychopathology (e.g., anxiety) was present, it was a requirement that this was secondary to chronic depersonalization, such that the latter was the predominant presenting symptom, and had been the dominant symptom for the duration of the condition (Medford et al., 2005). (2) On no psychotropic medication. Where patients had previously been on such medication, it was a study requirement that they had been medication-free for at least 6 months. However, toward the end of the study period, after four patients had dropped out of the study for various reasons, a decision was taken to include two further patients who were already on conventional antidepressant medication at time 1 (fluoxetine and venlafaxine, respectively). (3) Suitability for lamotrigine treatment, and having given informed consent for this treatment. (4) No contra-indication to MRI scanning, and willingness to undergo scanning on two separate occasions. (5) No history of other psychiatric or neurological illness.

All patients gave informed consent, and were told they were free to withdraw from the study at any time, without having to give any reason. Patients were further informed that declining to participate in, or withdrawing from, the study, would have no bearing on the nature or duration of their clinical care. The study had ethical approval from the relevant university and UK National Health Service review boards.

Fourteen patients with DPD satisfying entry criteria gave informed consent to participate (11 male, 3 female, age range 23–59, mean 33.7, SD 8.9). Estimated verbal IQ, assessed using the NART (National Adult Reading Test, Nelson, 1982) ranged from 103 to 127, mean 115, SD 7.79. Duration of DPD symptoms ranged from 2 to over 40 years, mean 15 years, SD 11.42. In other words all cases were highly chronic. Psychiatric symptoms were quantified using the Cambridge Depersonalization Scale (CDS, Sierra and Berrios, 2000), the Beck Depression Inventory (BDI, Beck et al., 1988), and the Spielberger Anxiety Inventory (SAI; Spielberger, 1983). The CDS is a self-report scale in which respondents are asked to rate the frequency and duration of depersonalization-related experiences over the preceding 6 months (Sierra and Berrios, 2000). Because we were interested in changes between two time points, rather than symptoms over a 6 month period, we used an adapted “state” version of the CDS, i.e., CDSS, comprising 22 depersonalization-related statements (e.g., *Things around me are now looking 'flat' or 'lifeless', as if I were looking at a picture*). Each statement is rated on a simple visual analog scale between 0 and 100, such that the maximum possible score is (22 × 100) = 2200. The BDI is a widely used instrument for probing mood state and other mental and behavioral aspects of relevance to depression, and SAI is another widely used anxiety scale that has two sections, one probing anxiety-related experiences over the preceding 6 months (Trait), and one asking respondents to rate feelings or experiences in the moment (State) (Spielberger, 1983).

Participants underwent the first fMRI scanning session prior to commencing treatment with lamotrigine, which was initiated at the dose of 25 mg per day and then slowly increased in fortnightly increments (Medford et al., 2005). Provided there were no adverse events related to treatment, they remained on this medication for a sufficient period to reach a target dose in the range 200–400 mg per day. It was not felt realistic or desirable to specify that all participants should be on the same target dose, as previous clinical experience with using lamotrigine for DPD strongly suggests that, when lamotrigine is effective, the dose at which it exerts optimal therapeutic effect varies widely between individuals (as is true for many drugs used to treat psychiatric conditions; Sierra et al., 2006).

Four patients reported that the DPD symptoms had originally arisen in the immediate aftermath of illicit drug use [cannabis in three cases, MDMA (“Ecstasy”) in the fourth, see Table 1]. It was not felt that this represented a significant confound, as those who described drug use preceding the onset of symptoms were otherwise inexperienced drug users who had avoided all illicit drugs since the first appearance of DPD symptoms. Moreover, in every case the episode of drug use was so far in the past as to make it very likely that, even if drug effects had played a role in the initial experience of depersonalization, the ongoing maintenance of the depersonalized state was due to psychological and biological processes underpinning DPD in general, rather than any distinct “drug-induced” variety of the condition. There is strong empirical evidence to support this assumption, from a study demonstrating that the course, nature and phenomenology of DPD is highly consistent irrespective of whether drug use was involved at onset (Medford et al., 2003).

**Table 1:** **Patients at time 1 and time 2: rating scale measures, medication at time 2, and improvement status (1 = improved, 2 = not improved)**.

	**Time 1**				**Medication (at time 2, daily dose)**	**Time 2**			**% change in CDSS score**	**Improved?**
	**BDI**	**SSAI**	**CDSS**	**Timelag (days)**		**BDI**	**SSAI**	**CDS (State)**		
1	1	26	475	180	Lamotrigine 400 mg	0	26	140	−70.5	1
2	27	58	780	242	Lamotrigine 400 mg	30	66	1175	+50.6	2
3	1	39	985	180	Lamotrigine 300 mg	3	31	615	−37.6	1
4	6	38	295	154	Lamotrigine 200 mg	10	46	285	−3.4	2
5	15	47	700	–	–	–	–	–	–	–
6	14	42	520	–	–	–	–	–	–	–
7	3	32	589	223	Lamotrigine 400 mg	4	37	237	−59.8	1
8	4	44	685	221	Lamotrigine 250 mg	4	55	344	−49.8	1
9	21	54	1355	–	–	–	–	–	–	–
10	10	57	591	–	–	–	–	–	–	–
11	6	29	467	127	Lamotrigine 250 mg	11	27	564	+20.8	2
12	18	50	1007	119	Lamotrigine 400 mg	17	53	1608	+59.7	2
					Venlafaxine 75 mg					
13	9	47	529	205	Lamotrigine 400 mg	9	46	588	+11.2	2
14	7	37	696	158	Lamotrigine 300 mg	5	22	260	−62.6	1
					Fluoxetine 20 mg					
Mean	10.14	42.86	691.00	180.9	–	9.3	40.9	581.6	–	–
SD	7.86	10.03	272.42	41.74	–	8.77	14.58	467.70	–	–

*Timelag, number of days between time 1 and time 2; BDI, Beck Depression Inventory; SSAI, Spielberger State Anxiety Inventory; DES, Dissociative Experiences Scale; CDS, Cambridge Depersonalization Scale (State Version). All 14 patients were scanned at time 1. Time 2 columns indicate which patients were subsequently re-scanned (10 of 14)*.

Of the original clinical sample of 14, four patients did not return for scans at time 2 (see Table 1). Two of these four withdrew from the study soon after their time 1 scans, deciding that they did not wish to take psychotropic medication. One patient experienced persistent nausea after commencing lamotrigine and stopped taking it after a week. The fourth patient took lamotrigine as described and then contacted the clinic to say she felt her DPD symptoms had been completely abolished by it, and that she no longer needed to be seen, and did not wish to attend for the second scan as she lived a considerable distance from the clinic.

At time 2, the 10 remaining patients were classified either as “improved” or “not improved” on the basis of clinical assessment and their scores on the Cambridge Depersonalization Scale, state version (CDSS, see Table 1). In keeping with previous work (Sierra et al., 2001, 2006), clinical improvement was defined as a reduction of at least 30% in CDSS score between time 1 and time 2, with percentage change calculated by the formula [(Time 1 CDSS score–Time 2 CDSS score)/Time 1 CDSS score] × 100. In every case, the categorisation as “improved” or “not improved” was corroborated by descriptions of symptoms and general mental state given by patients at clinical interview. Using this method, five of the 10 patients were classified as “improved” and five as “non-improved.” This 50% improvement rate is consistent with previous work examining lamotrigine as a potential treatment for DPD (Sierra et al., 2001, 2006). At this point it should be stressed that the current study does not represent a formal clinical trial of lamotrigine- aside from the small numbers, the study was not placebo-controlled or blinded, so the clinical improvement observed in some patients cannot be confidently ascribed to the effects of lamotrigine. Nevertheless, it is of note that placebo effects appear to be minimal in DPD (Sierra et al., 2001; Simeon et al., 2004), something particularly relevant here, given that patients had been experiencing symptoms for a prolonged period and in most cases had tried other psychotropic drug treatments (usually SSRIs) prior to referral to the DPD clinic, without any significant impact on their DPD symptoms.

In addition to the patient group, 25 healthy control subjects (14 male, 11 female) were also recruited (age range 23–46, mean 29.8, SD 5.68; estimated verbal IQ 107–127, mean 117.5, SD 5.47). Mean symptom scale scores for the control group were: CDSS 38.7 (SD 48.0), BDI 2.56 (2.73), SSAI 31.6 (SD 8.61).

Although the patient group contained relatively more males (11 M, 3 F vs. 14 M, 11 F), the difference in the gender composition of the two groups was not statistically significant (chi-square value 1.99, df = 1, *p* = 0.16). Unpaired *t*-tests were performed to check for other differences between the groups. There were no significant differences between groups for age (*t* = 1.66, df = 37, *p* = 0.11) or estimated verbal IQ (*t* = −1.18, df = 37, *p* = 0.24). There were highly significant differences between groups on the three clinical rating scales: the BDI (*t* = 4.41, df = 37, *p* < 0.001), the SSAI (*t* = 3.71, df = 37, *p* = 0.001), and the CDSS (*t* = 11.89, df = 37, *p* < 0.001).

### Experimental procedure and data analysis

Subjects underwent fMRI scanning during which they viewed alternating blocks of neutral and aversive images selected from the IAPS (Bradley and Lang, 1999). Each block of aversive or neutral images lasted 30 s and comprised five pictures, onscreen for 6 s each. Subjects were informed during the consent procedure that some images were potentially unpleasant or shocking, but were not given details as to the purpose of the experiment. For each image, subjects were asked to press a button to indicate whether the scene depicted was taking place outdoors or indoors. This simple task ensures cognitive processing of the stimulus image but does not involve explicit judgements of emotional arousal or salience: rather, the emotional processing is implicit. This is the same procedure used in our earlier fMRI study of depersonalization disorder utilizing IAPS images (Phillips et al., 2001).

During each scanning session, SCR was recorded continuously using standard silver-silver chloride electrodes 0.5 cm in diameter. Electrodes were attached to the first and second fingertips of the nondominant hand. All electrodes and cables were MR-compatible, allowing concurrent SCR recording during the fMRI scanning sessions. Using a skin conduction coupler, analog-to-digital conversion, and preamplification, the signal was processed by a PSYLAB (Cambridge, MA) SC5SA preamplifier, a SC5AL amplifier, and real-time PSYLAB online signal processing software. These devices employ a 24-bit A/D converter, filter the response signal at 10 Hz to prevent aliasing, and have an output range of 0–100 MicroSiemens. SCR was then sampled at 100 Hz without further compression and stored offline serially, including stimulus presentation points. Security measures were included to prevent electric shocks, radiofrequency (RF) burning, and to protect SCL signals from scanner-induced magnetic distortion using screened cables. Shielding of the wires received a 5.6-kOhm correction by resistors that produced a stable 1% shift of the signal. The cable was led from the magnetically shielded scanner environment through a Faraday cage, and the signal filtered at 1000 pF by capacitors to a copper cap before signal processing. Water soluble jelly (KY Jelly; Johnson and Johnson, Slough, England) was used as an electrolyte contact medium between electrodes and skin. Before the application of electrodes, all subjects washed their hands using a plain nonabrasive soap (as in Sierra et al., 2002) in order to standardize the dermo-gel-electrode interface across subjects as far as possible. During scanning, participants underwent approximately 12 min of structural scans prior to the commencement of the functional MRI experimental runs, allowing adequate time for habituation of SCR once in the scanning environment.

### fMRI hardware and data acquisition

Gradient echo echoplanar images were acquired on a GE Signa 1.5 T Neurovascular system (General Electric, Milwaukee, WI, USA) at the Maudsley Hospital, London. One hundred T2^*^-weighted images depicting BOLD (blood oxygenation level dependent) contrast (Ogawa et al., 1990) were acquired over 5 min (for each task) at each of 14 near-axial non-contiguous 5-mm thick planes parallel to the intercommissural (AC-PC) line: TE 40 ms, TR 3 s, in-plane resolution 5 mm, and interslice gap 0.5 mm. This EPI dataset provided complete coverage of the temporal lobes and almost complete coverage of the frontal, occipital, and parietal lobes (Simmons et al., 1999).

fMRI data were analyzed with the software XBAM, developed at the Institute of Psychiatry, using a non-parametric approach (see Fusar-Poli et al., 2010, for further discussion of this method and comparison with parametric mapping). Data were first processed (Bullmore et al., 1999a) to minimize motion related artifacts. A 3D volume consisting of the average intensity at each voxel over the whole experiment was calculated and used as a template. The 3D image volume at each timepoint was then realigned to this template by computing the combination of rotations (around the x, y, and z axes) and translations (in x, y, and z) that maximized the correlation between the image intensities of the volume in question and the template. Following realignment, data were then smoothed using a Gaussian filter (FWHM 7.2 mm) to improve the signal to noise characteristics of the images.

Responses to the experimental paradigms were then detected by first convolving each component of the experimental design with each of two gamma variate functions (peak responses at 4 and 8 s, respectively). The best fit between the weighted sum of these convolutions and the time series at each voxel was computed using a constrained BOLD effect model (Friman et al., 2003) This reduces the possibility of the model fitting procedure giving rise to mathematically plausible but physiologically implausible results. Following computation of the model fit, a goodness of fit statistic was computed. This consisted of the ratio of the sum of squares of deviations from the mean image intensity (over the whole time series) due to the model to the sum of squares of deviations due to the residuals (SSQratio). This statistic is used to overcome the problem inherent in the use of the F (variance ratio) statistic that the residual degrees of freedom are often unknown in fMRI time series due to the presence of colored noise in the signal. It has also been shown to behave equivalently to F under permutation testing (Edgington, 1995).

Following computation of the observed SSQratio at each voxel, the data are permuted by a wavelet-based method (Bullmore et al., 2001). Repeated application of this method at each voxel followed by recomputation of the SSQratio from the permuted data allows (by combination of results over all intracerebral voxels) the data-driven calculation of the null distribution of SSQratios under the assumption of no experimentally determined response. Using this distribution it is possible to calculate the critical value of SSQratio needed to threshold the maps at any desired type I error rate. In addition, detection of activated voxels is extended from voxel to cluster level (Bullmore et al., 1999b). In addition to the SSQratio, the size of the BOLD response to each experimental condition is computed for each individual at each voxel as a percentage of the mean resting image intensity level. In order to calculate the BOLD effect size, the difference between the maximum and minimum values of the fitted model for each condition is expressed as a percentage of the mean image intensity level over the whole time series. The observed and permuted SSQratio maps for each individual, as well as the BOLD effect size maps are transformed into the standard space (Talairach and Tournoux, 1988) using a two stage warping procedure (Brammer et al., 1997). This involves first computing the average image intensity map for each individual over the course of the experiment. The transformations required to map this image to the structural scan for each individual and then from “structural space” to the Talairach template are then computed by maximizing the correlation between the images at each stage. The SSQratio and BOLD effect size maps are then transformed into Talairach space using these transformations. Group activation maps are then computed by determining the median SSQratio at each voxel (over all individuals) in the observed and permuted data maps (medians are used to minimize outlier effects). The distribution of median SSQratios over all intracerebral voxels from the permuted data is then used to derive the null distribution of SSQratios and can be thresholded to produce group activation maps at any desired voxel or cluster-level type I error rate. Cluster level maps are thresholded at < 1 expected type I error cluster per brain. The computation of a standardized measure of effect SSQratio at the individual level, followed by analysis of the median SSQratio maps over all individuals treats intra and inter subject variations in effect separately, constituting a mixed effect approach to analysis.

Comparisons of responses between groups or experimental conditions were performed by fitting the data at each intraceberal voxel at which all subjects have non-zero data using a linear model of the type (Y = a + bX + e) where Y is the vector of BOLD effect sizes for each individual, X is the contrast matrix for the particular inter condition/group contrasts required, a is the mean effect across all individuals in the various conditions/groups, b is the computed group/condition difference and e is a vector of residual errors. The model is fitted by minimizing the sum of absolute deviations rather than the sums of squares to reduce outlier effects. The null distribution of b is computed by permuting data between conditions/groups (assuming the null hypothesis of no effect of experimental condition or group membership) and refitting the above model. Group difference maps are computed as described above at voxel or cluster level by appropriate thresholding of the null distribution of b. BOLD effect maps were used to compute significant group/condition differences rather than standardized measures such as SSQratio, F or t as these contain explicit noise components (error SSQ or error variance), raising the possibility that group differences resulting from F, SSQratio or t comparisons could reflect differences in noise rather than signal.

## Results

### Behavioral data

All subjects scored >95% correct responses when judging whether scenes depicted in IAPS images were taking place outdoors or indoors, with no difference between the groups.

### Skin conductance response (SCR) data

#### Group comparisons: all controls (n = 25) vs. DPD patients at time 1 (n = 14)

The following variables were examined: number of fluctuations (amplitude of over 0.02 microsiemens), amplitude of the largest fluctuation, and mean SCL (skin conductance level) across the epoch. These variables were computed for (i) the whole epoch (WE) (i.e., the entire scanning session), (ii) the first 30-s emotional block within each session (E1), and (iii) the first 30-s neutral block within each session (N1). As the experiment progresses, the likelihood of SCR responses in subsequent blocks being contaminated by responses to stimuli from earlier blocks increases. Therefore, SCR variables were not computed for individual blocks occurring after the first emotional and neutral blocks of each session. This resulted in a total of 9 SCR variables designated as WEflucs, WEamp, WEmean (whole epoch fluctuations, maximum amplitude, and mean SCR), E1flucs, E1amp, E1mean (the same variables computed for the first 30-s emotional block), and N1flucs, N1amp, N1mean (the same variables for the first 30-s neutral block).

Initial exploratory statistics performed on these nine variables, subdivided by group, showed that these data exhibited considerable variance that violated the assumptions of normality, even after log transformation. This was largely due to the data being positively skewed (e.g., four of the 14 DPD patients at time 1 showed no fluctuations greater than 0.02 microsiemens across the entire scanning epoch, meaning that for this group the distribution of values is skewed to the right of the normal distribution curve). For this reason, non-parametric tests were used to compare within and across groups.

Within the normal control group (*n* = 25), two-tailed Wilcoxon signed-rank tests were used to compare SCR responses in the first emotional and neutral blocks of each scanning epoch (E1 and N1, see above). Fluctuations in the initial emotional blocks (E1flucs) were significantly greater than those in initial neutral blocks (N1flucs; sum of ranks 34.5 for E1flucs, 118.5 for N1flucs, *p* = 0.041). There were no significant differences in amplitude of highest fluctuation or mean SCL. Similar results were obtained in the DPD patient group at time 1, *n* = 14, where E1flucs was significantly greater than N1flucs (sum of ranks 0 for E1flucs, 28.0 for N1flucs, *p* = 0.017), with no significant differences in mean SCL.

However, the key comparisons here are the group comparisons between controls and patients. Here, for each variable, group comparisons were performed using a Mann-Whitney U test, and in view of the relatively small sample sizes this statistic was corrected for exactness within SPSS. These data are summarized in Table 2. Tests of significance were one-tailed, as there was a clear *a priori* hypothesis that patients at time 1 would show diminished autonomic reactivity compared to controls. In the study by Sierra et al. (2002), the only measure on which DPD patients were found to be more reactive than controls was a shortened latency of response to non-specific stimuli (clap and sigh), while latency of response to aversive images of the type used in the current study was prolonged in the DPD patient group, and DPD patients also showed significantly reduced magnitude of SCR response to such pictures. Non-specific stimuli were not used in the current study, and latency of response was not computed as the methodology allows only for computation of responses across epochs or blocks, rather than response to individual events. For these reasons, the hypothesis for the current study was that at time 1, DPD patients would show consistently reduced autonomic activity (as indexed by SCR) compared to healthy controls, and this would be reflected across all variable types (number of fluctuations, amplitude, mean SCR level).

**Table 2:** **Group comparisons on SCR variables: controls vs. DPD patients (at time 1)**.

**SC Measure**	**Group**	**Mean rank**	**Sum of ranks**	***U***	***p***
WEflucs	NC	22.22	555.5	119.5	0.052
	DPDt1	16.04	224.5		
WEamp	NC	22.62	565.5	109.5	0.027^*^
	DPDt1	15.32	214.5		
WEmean	NC	22.24	556.0	119.0	0.052
	DPDt1	16.00	224.0		
E1flucs	NC	21.84	546.0	129.0	0.087
	DPDt1	16.71	234.0		
E1amp	NC	22.24	556.0	119.0	0.049^*^
	DPDt1	16.00	224.0		
E1mean	NC	22.28	557.0	118.0	0.049^*^
	DPDt1	15.93	223.0		
N1flucs	NC	23.70	592.5	82.5	0.002^*^
	DPDt1	13.39	187.5		
N1amp	NC	24.40	610.0	65.0	<0.001^*^
	DPDt1	12.14	170.0		
N1mean	NC	22.12	553.0	122.0	0.062
	DPDt1	16.21	227.0		

P-values corrected for exactness within SPSS. P-values marked

* *represent findings significant at a threshold of p = 0.05*.

For all subsequent SCR data analyses, non-parametric tests, corrected for exactness, were used, according to the rationale above.

All group differences were either significant at the <0.05 level or approaching that threshold.

#### Group comparisons: DPD patients at time 2, improvers (n = 5) vs. non-improvers (n = 5)

Here comparisons were made between improvers and non-improvers at time 2. There were no significant differences between the groups for any of the individual variables.

### Functional MRI data

#### Group data: controls (n = 25)

**Figure 1:**
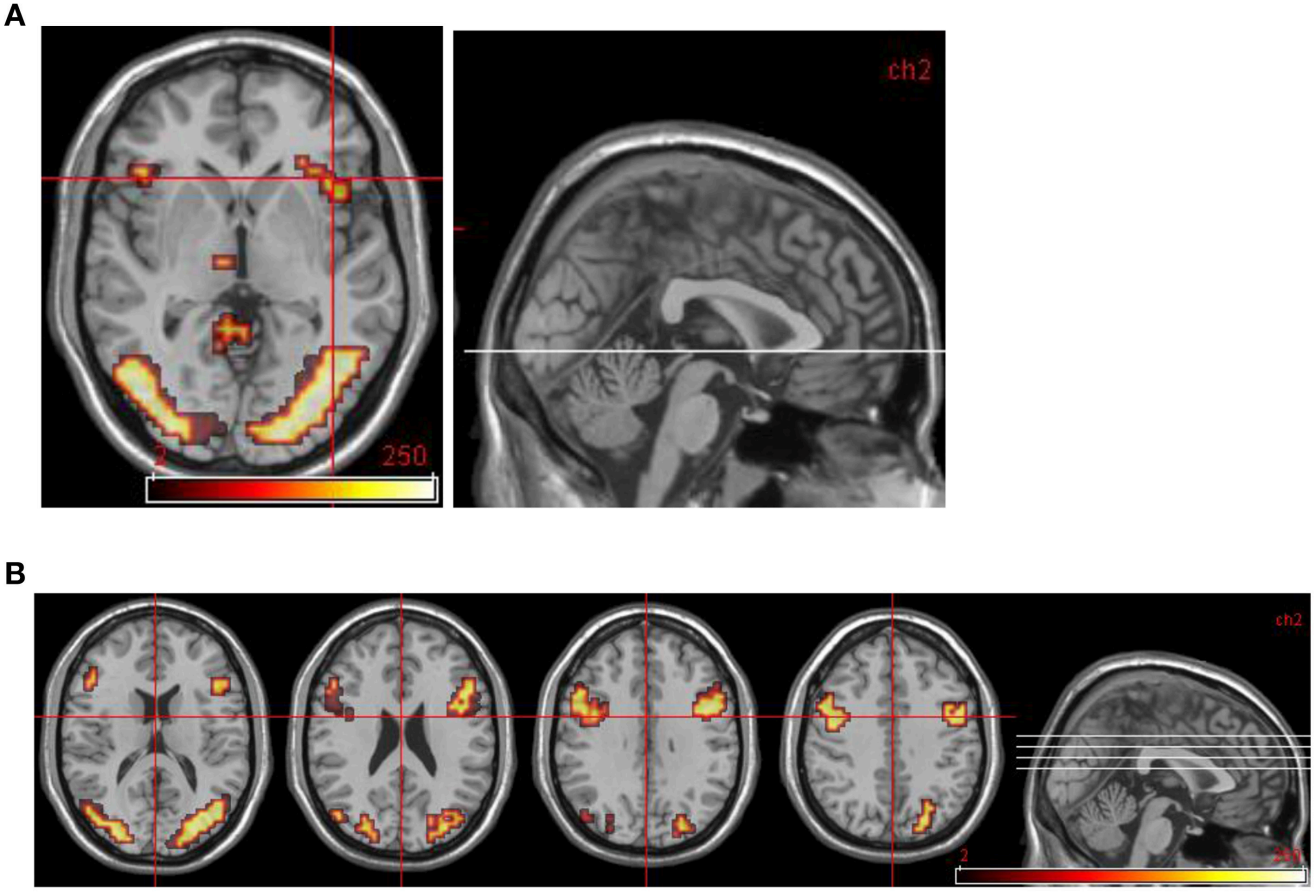
**Normal control group map. (A)** Transverse slice at *z* = −2 showing activation in R anterior insula. **(B)** Transverse slices at *z* = 16, 24, 32, 40 showing bilateral activation in DLPFC and occipital cortex. All areas significantly more activated by viewing aversive images compared to neutral images (i.e., in emotional phase).

For healthy controls, the key within-group analysis was the group-level comparison of regional brain activation in response to aversive images compared to neutral images. Areas significantly more activated by viewing aversive images were cerebellum, bilateral visual cortical areas, bilateral dorsolateral prefrontal cortex (DLPFC, Brodmann areas 44 and 45), right supramarginal gyrus. Areas significantly more activated by viewing neutral images were cerebellum, left middle temporal gyrus, right supramarginal gyrus, and left insula. These results are summarized in Table 3 (in all fMRI data tables that follow, for each cluster the Talairach co-ordinates of the maximally activated voxel within that cluster are shown) and Figure 1.

**Table 3:** **Areas of significantly greater activation in response to aversive images (upper part of table) and neutral images (lower part) in normal control subjects (***n*** = 25)**.

**No. of voxels**	***x***	***y***	***z***	**Region**	**Side**
**AREAS ACTIVATED BY EMOTIONAL** > **NEUTRAL**					
48	43	−59	−24	Cerebellum	R
37	36	−78	−13	BA18 Secondary visual cortex	R
25	−43	−70	−7	BA19 Secondary visual cortex	L
23	−40	−67	−29	Cerebellum	L
18	40	7	20	BA44 dorsolateral prefrontal cortex	R
14	−36	7	26	BA44 dorsolateral prefrontal cortex	L
10	40	22	−2	Anterior insula	R
8	51	−44	37	BA40 supramarginal gyrus	R
6	−51	19	20	BA45 dorsolateral prefrontal cortex	L
**AREAS ACTIVATED BY NEUTRAL** > **EMOTIONAL**					
66	−14	−52	−29	Cerebellum	L
45	−58	−22	−7	BA21 middle temporal gyrus	L
23	47	−52	37	BA40 supramarginal gyrus	R

*BA, Brodmann Area*.

#### Group data: DPD patients, time 1 (n = 14)

**Figure 2:**
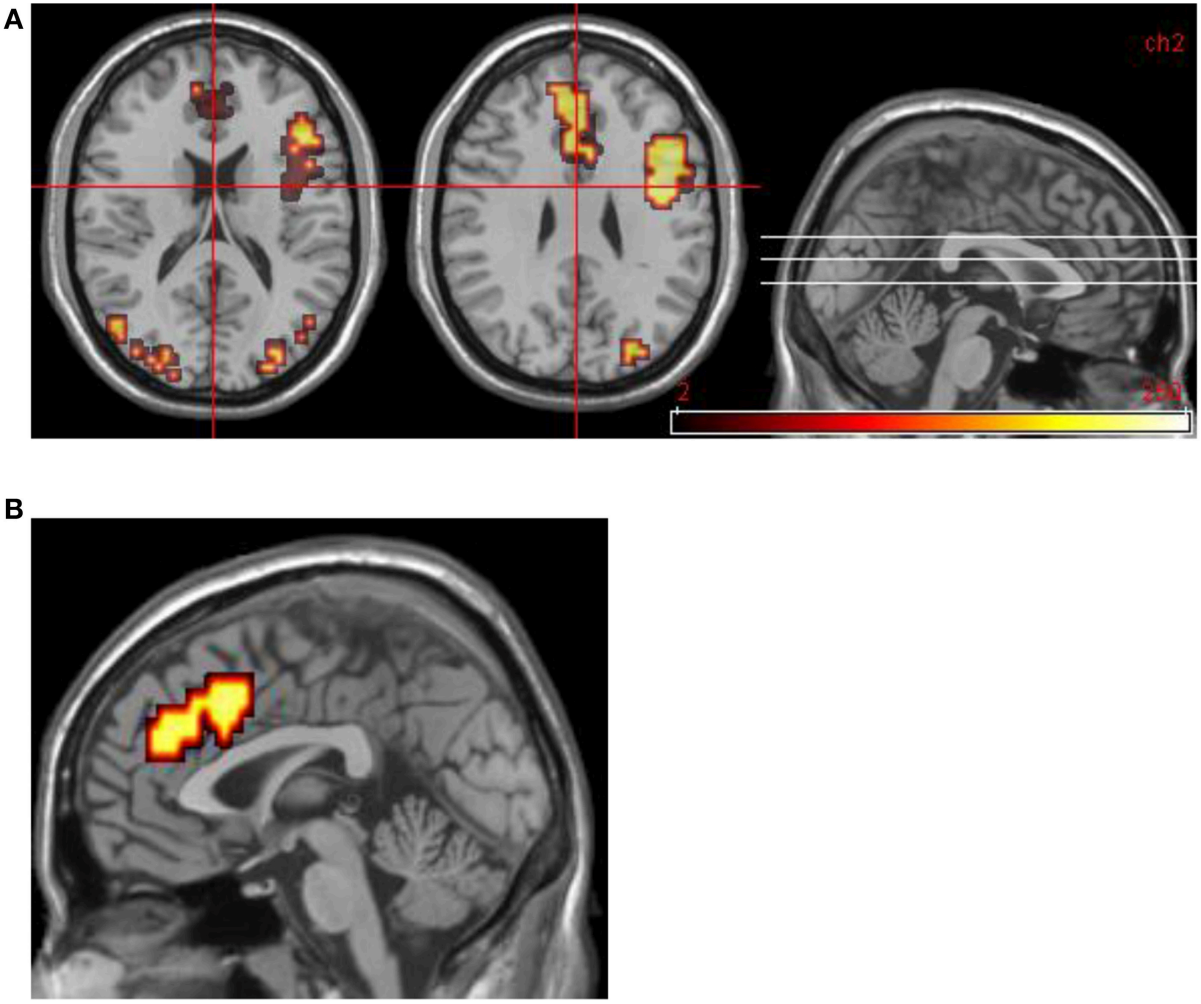
**Group data for all DPD patients at time 1. (A)** Transverse slices at level *z* = 18, 29 showing activations in emotional phase in bilateral visual cortex, right VLPFC, and bilateral medial prefrontal cortex (including ACC bilaterally). **(B)** Midline sagittal slice (i.e., x = 0) showing location of medial prefrontal activation.

Areas significantly more activated by viewing aversive images were areas of right lateral prefrontal cortex, bilateral primary visual cortex, bilateral anterior cingulate cortex (ACC), and left medial prefrontal cortex. Middle temporal gyrus was significantly activated by viewing neutral images in contrast to aversive images (see Table 4 and Figure 2).

**Table 4:** **Areas of significantly greater activation in response to aversive images (upper part of table) and neutral images (lower part) in patients with DPD at time1 (***n*** = 14)**.

**Size**	***x***	***y***	***z***	**Region**	**Side**
**AREAS ACTIVATED By EMOTIONAL** > **NEUTRAL**					
38	43	7	20	BA44 DLPFC	R
36	43	−70	7	BA37 primary visual cortex	R
32	0	22	26	BA 24/32 anterior cingulate cortex	R
29	−4	26	20	BA24 anterior cingulate cortex	L
28	40	4	26	BA44 DLPFC	R
27	43	19	15	BA 44/45 DLPFC	R
21	0	22	31	BA32 anterior cingulate cortex	R
17	−43	−70	−2	BA19/37 primary visual cortex	L
15	40	30	−7	BA47 VLPFC	R
13	−7	52	15	BA9 medial prefrontal cortex	L
11	40	30	9	BA 45/46 DLPFC	R
**AREAS ACTIVATED BY NEUTRAL** > **EMOTIONAL**					
224	58	−15	−13	BA21 middle temporal gyrus	R

*BA, Brodmann Area; DLPFC, dorsolateral prefrontal cortex*.

#### Group data: DPD patients at time 2, improvers (n = 5)

**Figure 3:**
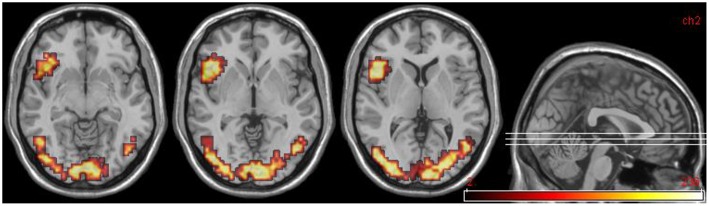
**DPD patients at time 2: group data for improvers (n = 5) only**. Transverse slices at levels *z* = −8, −2, 6, showing activations in emotional phase in visual cortex bilaterally, and left anterior insula extending into DLPFC.

Areas significantly more activated by viewing aversive images were areas of bilateral visual cortex, left DLPFC, and left anterior insula (Table 5, Figure 3). One large cluster in cerebellum was significantly more active in the neutral phase.

**Table 5:** **Areas of significantly greater activation in response to aversive images and neutral images in those DPD patients who at time 2 exhibited a significant decrease in CDSS score (***n*** = 5)**.

**Size**	***x***	***y***	***z***	**Region**	**Side**
**AREAS ACTIVATED BY EMOTIONAL** > **NEUTRAL**					
45	22	−81	−7	BA18 visual cortex	R
38	29	−63	−29	Cerebellum	R
37	−22	−74	−29	Cerebellum	L
32	−7	−89	−13	BA17 lingual gyrus/visual cortex	L
29	−43	19	−7	Anterior insula/BA47	L
26	−51	19	9	BA45	L
19	−32	30	4	Anterior insula	L
11	−47	19	20	BA45 DLPFC	L
9	−40	0	31	BA44 DLPFC	L
**AREAS ACTIVATED BY NEUTRAL** > **EMOTIONAL**					
455	14	−52	−29	Cerebellum	R

#### Group data: DPD patients at time 2, non-improvers (n = 5)

**Figure 4:**
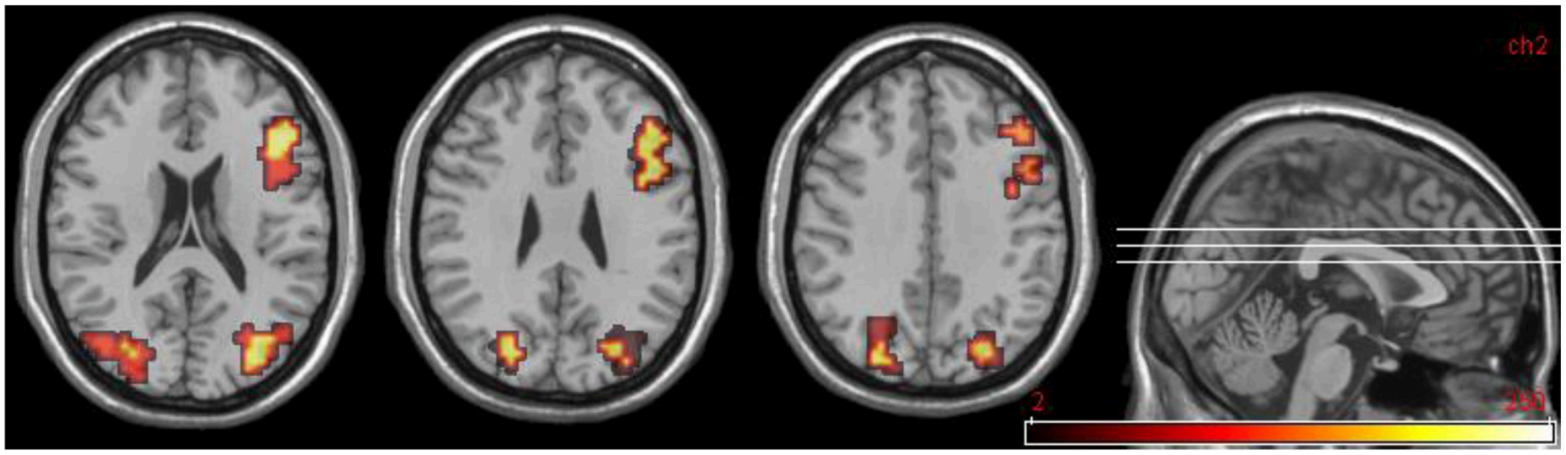
**DPD patients at time 2, group data for non-improvers (***n*** = 5)**. Transverse slices at *z* = 20, 28, 36 showing activations in emotional phase in bilateral visual cortex and right DLPFC.

Areas significantly more activated by viewing aversive images were areas of bilateral visual cortex, right DLPFC, right VLPFC (Brodmann area 47), and cerebellum. For the reverse comparison, one large cluster in cerebellum was observed (Table 6, Figure 4).

**Table 6:** **Areas of significantly greater activation in response to aversive images and neutral images in those DPD patients who at time 2 did not exhibit a significant decrease in CDSS score (***n*** = 5)**.

**Size**	***x***	***y***	***z***	**Region**	**Side**
**AREAS ACTIVATED BY EMOTIONAL** > **NEUTRAL**					
74	36	−67	−18	Cerebellum	R
64	51	−63	−13	BA19/37 visual cortex	R
40	−40	−67	−24	Cerebellum	L
36	47	−67	−2	BA19 visual cortex	R
34	−43	−70	−7	BA19/37 visual cortex	L
26	43	30	15	BA45, DLPFC	R
14	43	30	−7	BA47, VLPFC	R
**AREAS ACTIVATED BY NEUTRAL** > **EMOTIONAL**					
87	−25	−33	−29	Cerebellum	L

#### Group comparisons: controls (n = 25) vs. all DPD patients at time 1 (n = 14)

**Figure 5:**
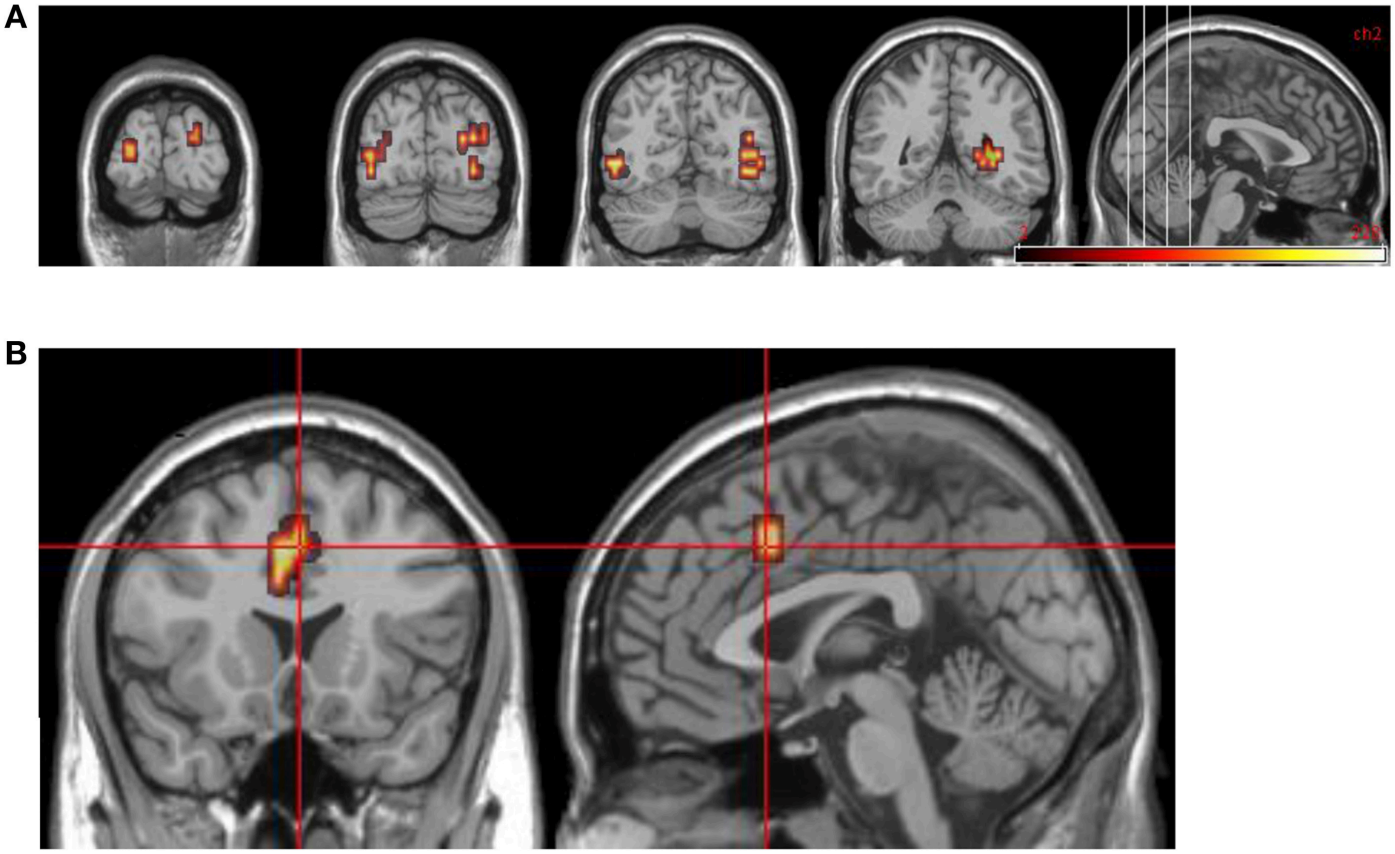
**Group comparisons between controls and DPD patients at time 1. (A)** Areas significantly more active in emotional phase in controls: coronal slices showing extensive bilateral visual cortical activity. **(B)** Areas significantly more active in DPD patients: coronal and midline sagittal views showing location of anterior cingulate cortex activation.

Findings from the controls vs. patients (at time 1) comparison are summarized in Table 7 and Figure 5.

**Table 7:** **Comparison between controls and DPD patients at time 1**.

**Size**	***x***	***y***	***z***	**Region**	**Side**	***p***
**CONTROLS** > **DPD PATIENTS**						
70	47	−63	−2	BA19 2viscx	R	0.005
55	−43	−67	−13	BA19 2vis cx	L	0.006
**DPD PATIENTS** > **CONTROLS**						
27	40	4	20	BA44 DLPFC	R	0.037
26	0	22	31	BA32 ACC	R/L	0.039

*All activations shown were in the emotional phase. 2vis cx, secondary visual cortex; DLPFC, dorsolateral prefrontal cortex; ACC, anterior cingulate cortex*.

#### Group comparisons: DPD patients at time 2, improvers (n = 5) vs. non-improvers (n = 5)

**Figure 6:**
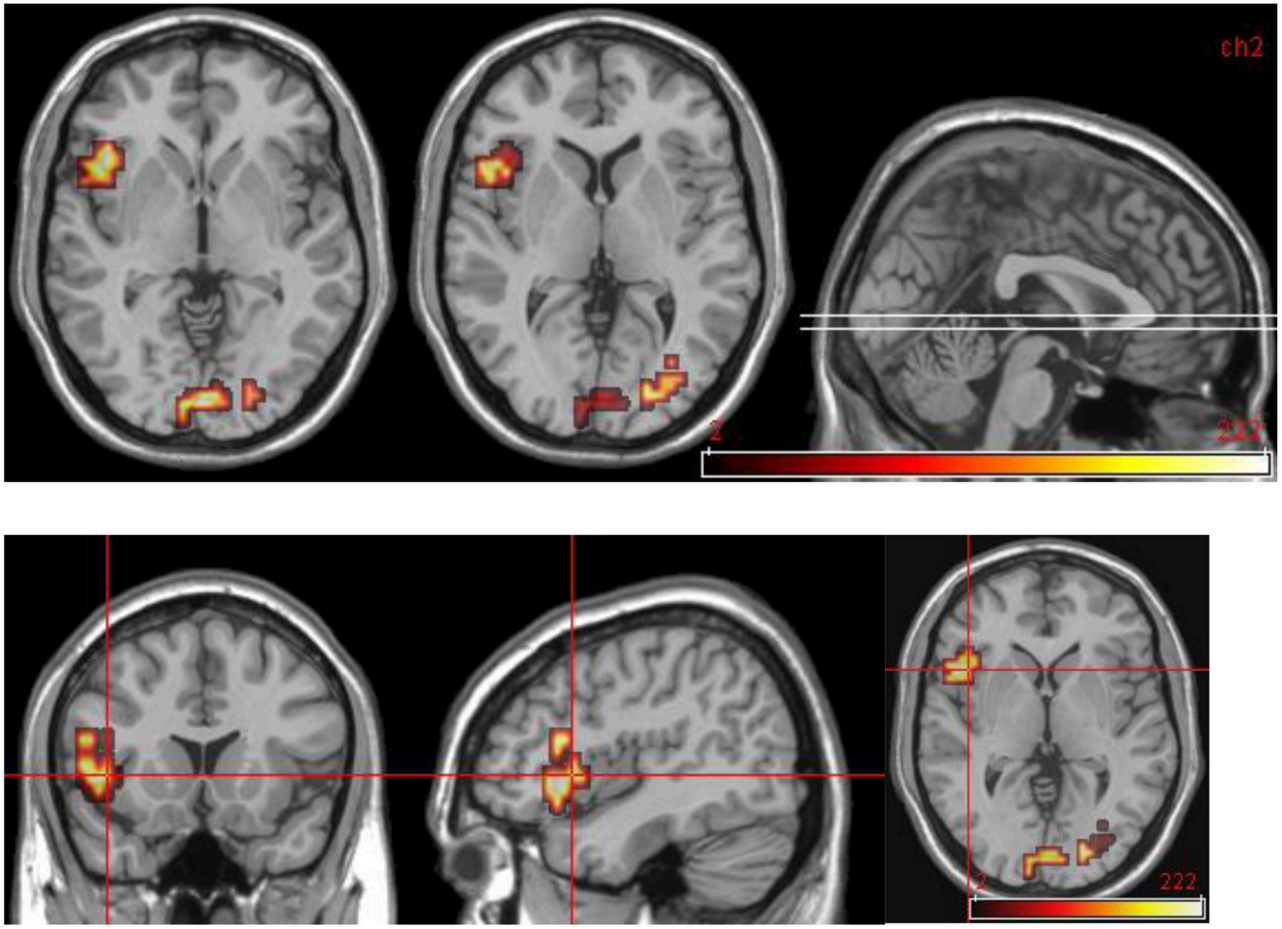
**Areas significantly more activated in emotional phase in improvers compared to non-improvers at time 2**. Crosshair projection centered on left anterior insula.

For this comparison, time 2 data for DPD patients who showed a clinically significant reduction in CDSS score (*n* = 5) was compared with time 2 data for those patients who did not show any such reduction. See Table 8 and Figure 6.

**Table 8:** **DPD patients: comparison between improvers and non-improvers at time 2**.

**Size**	***x***	***y***	***z***	**Region**	**Side**	***p***
**AREAS ACTIVATED IN IMPROVERS** > **NON-IMPROVERS**						
55	−43	19	−7	Anterior Insula	L	0.013
50	−7	−89	−18	Cerebellum	L	0.016
25	32	−74	4	BA19 visual cortex	R	0.029
**AREAS ACTIVATED IN NON-IMPROVERS** > **IMPROVERS**						
101	14	−52	−29	Cerebellum	R	0.003
15	18	−85	−12	BA18 visual cortex	R	0.042
12	−36	−74	4	BA19 visual cortex	L	0.026

*All activations shown were in the emotional phase. 2vis cx, secondary visual cortex*.

#### Comparison between improvers at time 1 and time 2

**Figure 7:**
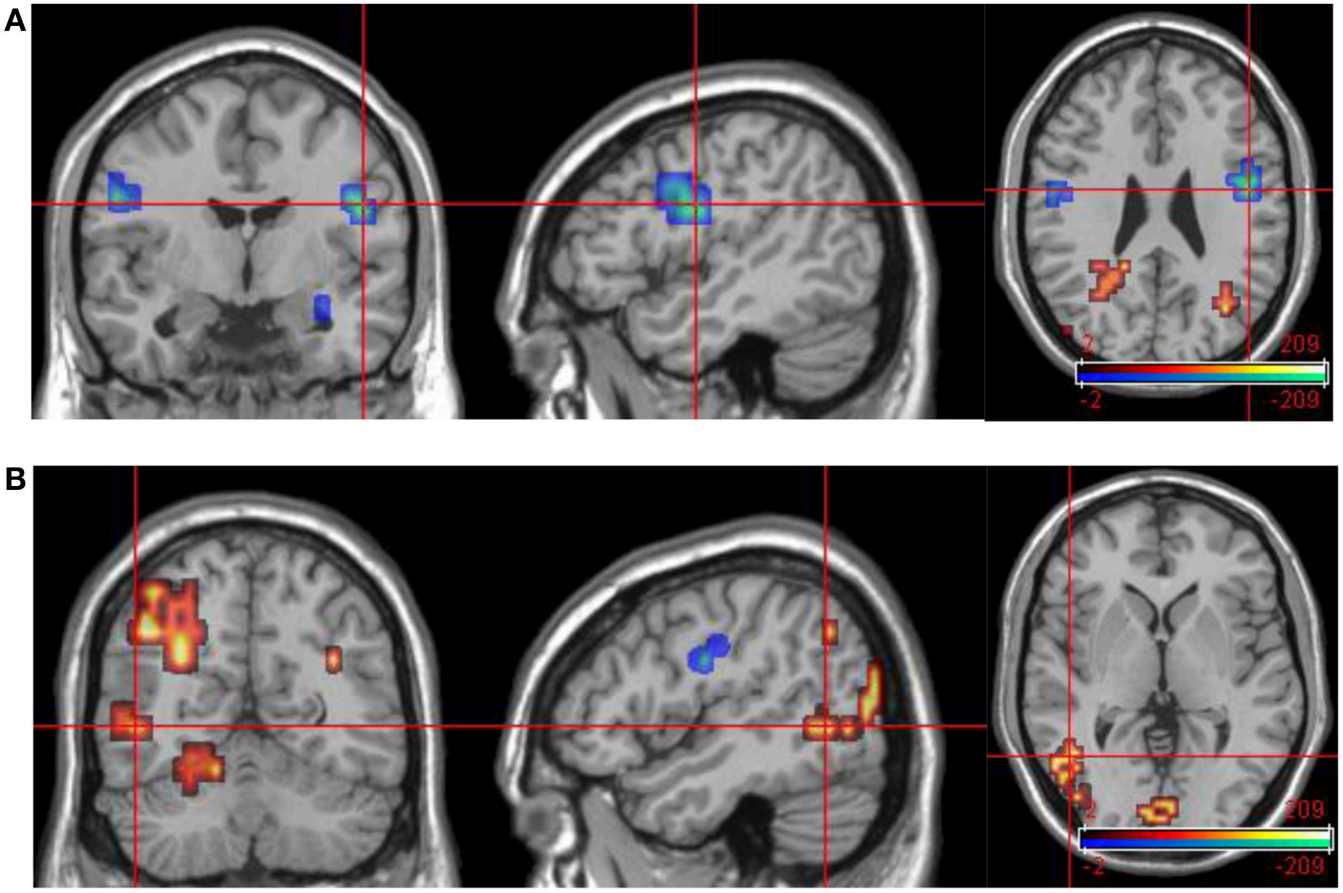
**Group data comparisons for improvers at time 1 and time 2**. Areas significantly more active in emotional phase at time 1 shown in fire, areas significantly more active at time 2 in blue. **(A)** Transverse slice at *z* = 26 with crosshair projection centered on cluster in R DLPFC. Note activation in similar contralateral region. **(B)** Extensive posterior cortical activation at time 2 (> time 1). Transverse slice at *z* = 1, crosshair projection centered on cluster in L occipitotemporal cortex.

These data are summarized in Table 9 and Figure 7.

**Table 9:** **DPD patients who showed a significant fall in CDSS score compared at time 1 and time 2**.

**Size**	***x***	***y***	***z***	**Region**	**Side**	***p***
**TIME 2** > **TIME 1**						
57	−26	−59	31	BA19 1vis cx	L	0.008
34	−7	−70	−29	Cerebellum	L	0.007
19	36	−63	31	BA19 1vis cx	R	0.019
14	−47	−63	−7	BA18/37 2vis cx	L	0.030
**TIME 1** > **TIME 2**						
21	47	4	15	BA44 DLPFC	R	0.013
12	29	−26	−29	Cerebellum	R	0.039
12	−51	−7	26	BA4 precentral gyrus	L	0.036

*Abbreviations as above*.

## Discussion

With regard to the rating scale data: it is unsurprising that patients and controls differed significantly on the anxiety scale (SSAI) in view of the established relationship between depersonalization and anxiety (Medford et al., 2005, although DPD is not simply an anxiety disorder, see Sierra et al., 2012), and similar anxiety scores have been found in previous empirical studies of patients with primary DPD (e.g., Sierra et al., 2002). With regard to scores on the BDI, although DPD patients scored significantly higher than controls, it should nevertheless be noted that within the patient group the mean score is still well below the usual clinical cut-off, as are all the individual scores within the patient group, with the exception of one individual scoring 30 and another with a borderline score of 17.

SCR data was analyzed along three axes (number of fluctuations, amplitude of highest fluctuation, and mean SCR level) for the whole scanning session and, in addition, for the first 30-s aversive and neutral stimulus blocks, giving a total of nine dependent variables. Tests of the hypothesis that DPD patients (at time 1) would be generally less autonomically reactive than healthy controls produced significant results for five of the nine variables, and non-significant trends in the same direction for the other four variables. Taken as a whole, these findings provide strong evidence to support the above hypothesis. It should be noted that this effect was not specific to responses to emotional stimuli: the findings from neutral blocks are similar to those from emotional blocks. As discussed above, the study by Sierra et al. (2002), found decreased latency of response to physical (non-image) auditory stimuli (clap, sigh) in the DPD patient group compared to controls. The authors interpreted this as supporting a previous theoretical suggestion (Sierra and Berrios, 1998) that in DPD there is an inhibition of emotional response combined with a simultaneous state of heightened alertness or vigilance (this latter aspect was not specifically examined in the current study). However, in the same study, in addition to the finding that DPD patients had diminished responsiveness to aversive images, there was also weak evidence (not reaching statistical significance) of diminished responses to neutral images in the DPD patient group.

There is also older work supporting the idea that depersonalization is associated with diminished emotional arousal and autonomic reactivity. There are reports of individuals with episodic depersonalization in whom periods of depersonalization were associated with SCR response patterns that were strikingly different (in terms of showing greatly reduced amplitude, and lack of fluctuation) from SCR responses recorded from the same individuals at times when they were not experiencing depersonalization symptoms (Lader and Wing, 1966). Another study (Kelly and Walter, 1968) used forearm blood flow as an index of sympathetic activity and found that patients with depersonalization had the lowest baseline levels compared to various other psychiatric patient groups. Thus the finding of reduced autonomic reactivity in depersonalization is a consistent one, and in line with the results of the current study.

Improvements in clinical state at time 2, as indexed by CDSS score, were not, however, associated with any significant change in SCR variables. No significant differences were seen in individual SCR variables between improvers and non-improvers at time 2, or when comparing SCR results in improvers at time 1 and time 2. Caution must be exercised in interpretation of these findings, as these comparisons are between small groups (two groups of five in each case). Keeping this caveat in mind, it appears that while measures of SCR reactivity appear to reliably discriminate patients from controls, they do not appear to have predictive value in terms of response to pharmacotherapy or progress over time, and nor are improvements in clinical state necessarily mirrored by corresponding changes in autonomic responses. This may reflect the fact that at time 2, even those patients who exhibited a significant fall in CDSS score compared to time 1 nevertheless still had CDSS scores well above the clinical cut-off for this scale, with only one exception. In other words, four of these five patients still had definite symptoms of DPD, despite having improved clinically to a significant degree. The improvement appears to be reflected in changes in neural activation pattern in response to the IAPS images (discussed in detail below), but this is not mirrored by changes in autonomic response. This suggests that the inhibition of autonomic emotional response is a core feature of the DPD syndrome which is one of the last aspects to “normalize” when patients improve clinically, whether this improvement is spontaneous or in response to specific treatment interventions. Although patients who improved were designated as “improvers” for the purposes of this study, it cannot be definitively stated that their improvement was in fact due to the prescribed pharmacotherapy. The study was not designed as a formal treatment trial- rather it is an observational study of changes over time in a group of patients, and the cause of these changes cannot be definitely inferred. However, the response rate of 50% (among the 10 patients who were re-scanned at time 2) is consistent with a previous study of lamotrigine (either as monotherapy or in combination with antidepressant medication) (Sierra et al., 2006), and it should also be noted that all the patients had at time 1 reported chronic, intractable symptoms that had not responded to any previous interventions.

In contrast to the SCR data, the functional MRI results suggest a clear pattern of association between changes in mental state over time and corresponding changes in regional brain activity. At time 1, DPD patients showed significant activation in response to aversive images in right lateral prefrontal cortex (both ventrolateral and dorsolateral regions), bilateral primary visual cortex, bilateral ACC, and left medial prefrontal cortex. Visual cortical activations are frequently seen in functional neuroimaging studies when emotionally salient material is presented in the visual modality, as in the current study. This is thought to reflect the “modulation” of sensory cortex by back projections from areas involved in emotional processing (e.g., Morris et al., 1998; Tabbert et al., 2005). This process appears to still be in operation in the DPD patients, despite their otherwise reduced biological and experiential response to emotion. However, it is important to note that this modulatory effect appears to increase when symptoms of depersonalization are (at least partly) ameliorated. The evidence for this is that when group data for improvers at time 2 was contrasted with equivalent data from time 1 for the same subgroup of patients, there was significantly more activation in visual cortical areas (primary visual cortex bilaterally, and left secondary visual cortex) at time 2. This suggests that the modulatory effect seen in visual cortex at time 1 is less pronounced than would normally be expected, and this is confirmed by the fact that at time 1, controls showed significantly greater activation than DPD patients in visual cortex bilaterally. These activations were large (70 and 55 voxels, respectively) and highly statistically significant (*p* < 0.01). In contrast, no visual cortical areas were more active in DPD patients than in controls. However, in DPD patients at time 2, the pattern of results in visual cortex is more complex. Both improvers and non-improvers show bilateral visual cortical activations at time 2, with different areas being significantly more active in each of these two subgroups. However, it is of note that in the responder subgroup, visual cortex was significantly more active at time 2 than at time 1, suggesting that a reduction in CDSS score is associated with increased modulation of sensory cortex by emotional processing. It may be that as this modulatory effect increases, different areas of secondary sensory cortex are recruited, perhaps accounting for the different patterns of visual cortical activity seen in the two subgroups.

A network of right prefrontal regions was activated by viewing aversive images in the DPD patient group at time 1. This included a specific region (falling within Brodmann area 47) of right ventrolateral prefrontal cortex. In a previous fMRI study of DPD (Phillips et al., 2001), this same region was also found to be preferentially activated in DPD patients in response to aversive images compared to neutral images, and it was suggested that this region may have a key role in an involuntary “top-down” inhibition of brain regions involved in the generation of emotional response. The current study provides further evidence that this area plays a key role in the biological underpinnings of the depersonalization state. Firstly, it was activated in response to aversive images in patients at time 1, as detailed above. Secondly, the same area was activated by viewing aversive images in non-improvers at time 2, but not in improvers. This suggests that lessening in the severity of depersonalization symptoms is associated with reduced activity in this area during emotional processing. The implication of this is that right BA47 is a critical region for the inhibition of emotional responses in DPD. In this context, it is interesting to note that the same region has repeatedly been identified as an important inhibitory area in studies that have examined voluntary regulation of emotion in normal volunteers (Ochsner and Gross, 2005; Ohira et al., 2006). Taken together, these findings suggest that right BA47 is recruited when emotional responses are inhibited, regardless of whether this emotional inhibition is volitional (as in normal voluntary emotional self-regulation) or not (as in DPD).

Other lateral prefrontal regions activated in DPD patients by viewing aversive images were predominantly right-sided, with BA44, 45, and 46 all involved. An area of right DLPFC was significantly more active in DPD patients at time 1 compared to controls. This suggests that, in addition to the area of right VLPFC detailed above, more dorsal regions of the right lateral prefrontal cortex are also involved in the inhibition of emotional response formation in DPD.

Medial prefrontal areas, including bilateral ACC, were also activated by viewing aversive images in DPD patients at time 1. ACC is known to be a key area in the evaluation of emotional salience and regulation of emotional response (Medford and Critchley, 2010) and it might be seen as a candidate for an important role in the inhibition of emotional responses in DPD. The current study provides only partial support for this idea. ACC was activated in patients at time 1, but ACC activation was not seen in either patient subgroup (improvers or non-improvers) at time 2, nor did ACC emerge as an area of significant difference between the two subgroups at time 2.

One possible objection to our study might be that the time 2 scan results could have been confounded by some effect of lamotrigine on regional brain perfusion and therefore on BOLD response, as there is one study suggesting that lamotrigine may reduce the BOLD response to somatosensory stimulation in rodents (Kida et al., 2006). More pertinent to the current work, however, is a more recent study showing no significant effect of lamotrigine on brain perfusion patterns in humans (Shcherbinin et al., 2015), suggesting that any such confound is unlikely. Furthermore, in a previous fMRI study of DPD in which some patients were on lamotrigine and some were unmedicated, subgroup analyses of unmedicated participants did not suggest a confounding effect of lamotrigine (Medford et al., 2006).

The left anterior insula emerged as a key region in this study. This region was activated in controls in response to aversive images, and this finding is consistent with a large body of literature implicating the anterior insula in the response to emotionally salient (particularly aversive) material (Phillips et al., 1997; Mataix-Cols et al., 2008; Meriau et al., 2009). Insula activation was not seen in response to emotional images in DPD patients at time 1. However, anterior insula was activated by viewing aversive images in improvers at time 2, but not in non-improvers at time 2, and the difference was statistically significant when the two groups were compared. This provides powerful evidence that a lack of anterior insula activity is related to the diminished emotional responsiveness seen in DPD, and that a “re-awakened” insula is seen when patients improve and de-affectualization symptoms (and DPD symptoms more generally) are ameliorated.

The insula is frequently reported as a key brain region in processes relating to emotion and bodily sensation (Craig, 2009). In particular it has received attention for its putative role in interoception: “our ability to sense ourselves” (Damasio et al., 2000; Craig, 2002, 2009; Damasio, 2003; Critchley et al., 2004; Medford and Critchley, 2010). Interoception encompasses the sensing of discrete bodily events such as heartbeats, in addition to a more general sense of bodily state thought to rely largely on sensation related to autonomic and visceromotor processes (Farb et al., 2015). It is widely argued that this general feeling state is closely related to emotional state (Craig, 2002, 2009; Medford and Critchley, 2010). This relationship of interoceptive processes to emotional state, in both healthy and clinical groups, has been the focus of considerable empirical and theoretical work in recent years (for recent reviews see Barrett and Simmons, 2015; Farb et al., 2015). One influential conceptualization suggests that ascending somatosensory information is collated and integrated (“represented”) in posterior and mid insula (regarded as “primary interoceptive cortex”) before then being “re-represented” by anterior insula to generate a consciously accessible feeling state (“how I feel”) (Craig, 2009): a “sense of the whole” or “sense of the internal milieu.” Theorists differ according to how much of a central role they attribute to the insula. For example it has been suggested (Craig, 2009) that the insula may be the seat of self-awareness itself, whereas a more recent formulation (Barrett and Simmons, 2015) argues that insular cortex is rather one of a number of interoceptive hubs, so that while it contributes to interoception, it is not crucial for it. Certainly the former, more dramatic, claim appears inconsistent with the fact that case studies of patients with extensive bilateral insula damage (Philippi et al., 2012; Damasio et al., 2013; Feinstein et al., 2015) show that fundamental aspects of self-awareness (such as the ability to recognize oneself, or to have apparently normal experiences of agency) and emotional response are preserved in such cases. However, the disturbance of self-awareness encountered in DPD is of a more subtle type: patients retain basic self-awareness, and do not become delusional, rather something about the quality of self-experience has changed. Patients with DPD will often use the phrase “.as if” when attempting to convey their experiences of the depersonalized state (e.g., “. *as if* I were an automaton,” “. *as if* the world were artificial”), a state in which experience of both the self and the surrounding world feel oddly attenuated. Thus work in DPD may provide a clinical sounding-board for the notion that, while insular cortex may not be essential for interoceptive experience, its activity is important in some way that adds important higher-order experiential features (Damasio et al., 2013), aspects of subjective experience that can usually be taken for granted but which are compromised in DPD.

It is interesting that in our sample, DPD patients showing clinical improvement at time 2 showed no significant change in their sympathetic arousal as indexed by skin conductance. This suggests that their self-reported improved self-awareness, and concomitant changes in neural activity, may not be immediately related to changes in sympathetic arousal but rather to other interoceptive changes—here it is worth recalling that the bodily signals that contribute to interoceptive experience are manifold, including autonomic, visceromotor, hormonal, immunological, and humoral elements (Critchley and Harrison, 2013). With regard to interoception and DPD, a study of heartbeat awareness in DPD found no difference in performance between DPD patients and healthy controls (Michal et al., 2014), but more recent work suggests some anomalies in the cortical representation of bodily events in DPD (Schulz et al., 2015). In any event, in DPD the alteration in self-experience may not be best probed by studies measuring awareness of discrete events such as heartbeats, as patient self-reports strongly suggest that in DPD it is a more general sense of one's physical being that is somehow attenuated or compromised (Medford, 2012).

In this context, it is of particular interest that the insula should emerge as critical in DPD: in view of its role in the generation of feeling states, and the relationship between these and emotional experience, diminished (or inhibited) anterior insula activity is a plausible biological substrate for both desomatization symptoms and the phenomenon of de-affectualization which is the explicit target of this study. It may be, then, that the subjective alteration of experience that is the core of the depersonalized state is rooted in a lack of anterior insula activity. The fact that this lack of activity appears to be amenable to treatment- or, at least, that it is not fixed over time- is encouraging, as it suggests a potential neurobiological target for future treatment strategies in DPD.

## Author contributions

NM recruited most of the participants, designed and ran the fMRI experiments, carried out the bulk of the data analysis and interpretation, prepared the figures, and wrote most of the manuscript. MS recruited the remaining participants and contributed to theoretical discussion of the findings. AS contributed to data analysis and theoretical discussion. VG and MB contributed to the analysis both theoretically (writing software routines) and practically (helping to write the Methods section). AD supervised the work and contributed to the conceptual background and theoretical discussion.

## Funding

NM was supported by a Wellcome Trust Research Training Fellowship, ref 065799/Z/01/Z.

### Conflict of interest statement

The authors declare that the research was conducted in the absence of any commercial or financial relationships that could be construed as a potential conflict of interest.

## References

[B1] Barrett L. F. Simmons W. K. (2015). Interoceptive predictions in the brain. Nat. Rev. Neurosci. 16, 419–429. 10.1038/nrn3950 26016744 PMC4731102

[B2] Beck A. Steer R. Garbin M. (1988). Psychometric properties of the Beck Depression Inventory: twenty-five years of evaluation. Clin. Psychol. Rev. 8, 77–100. 10.1016/0272-7358(88)90050-5

[B3] Bradley M. M. Lang P. J. (1999). Affective Norms for English Words (ANEW). Gainesville, FL: NIMH Center for the Study of Emotion and Attention, University of Florida.

[B4] Brammer M. J. Bullmore E. T. Simmons A. Williams S. C. R. Grasby P. M. Howard R. J.. (1997). Generic brain activation mapping in functional magnetic resonance imaging: a nonparametric approach. Magn. Reson. Imaging 15, 763–770. 10.1016/S0730-725X(97)00135-5 9309607

[B5] Bullmore E. T. Brammer M. J. Rabe-Hesketh S. Curtis V. A. Morris R. G. Williams S. C. R.. (1999a). Methods for diagnosis and treatment of stimulus-correlated motion in generic brain activation studies using fMRI. Hum. Brain Mapp. 7, 38–48. 9882089 10.1002/(SICI)1097-0193(1999)7:1<38::AID-HBM4>3.0.CO;2-Q PMC6873318

[B6] Bullmore E. T. Long C. Suckling J. Fadili J. Calvert G. Zelaya F. (2001). Colored noise and computational inference in fMRI time series analysis: resampling methods in time and wavelet domains. Neuroimage 13, S86 10.1016/S1053-8119(01)91429-6 PMC6871881 11169871

[B7] Bullmore E. T. Suckling J. Overmeyer S. Rabe-Hesketh S. Taylor E. Brammer M. J. (1999b). Global, voxel, and cluster tests, by theory and permutation, for a difference between two groups of structural MR images of the brain. IEEE Trans. Med. Imaging 18, 32–42. 10.1109/42.750253 10193695

[B8] Craig A. D. (2002). How do you feel? Interoception: the sense of the physiological condition of the body. Nat. Rev. Neurosci. 3, 655–666. 10.1038/nrn894 12154366

[B9] Craig A. D. (2009). How do you feel–now? The anterior insula and human awareness. Nat. Rev. Neurosci. 10, 59–70. 10.1038/nrn2555 19096369

[B10] Critchley H. D. Harrison N. A. (2013). Visceral influences on brain and behavior. Neuron 77, 624–638. 10.1016/j.neuron.2013.02.008 23439117

[B11] Critchley H. D. Wiens S. Rotshtein P. Ohman A. Dolan R. J. (2004). Neural systems supporting interoceptive awareness. Nat. Neurosci. 7, 189–195. 10.1038/nn1176 14730305

[B12] Damasio A. (2003). Feelings of emotion and the self. Ann. N.Y. Acad. Sci. 1001, 253–261. 10.1196/annals.1279.014 14625365

[B13] Damasio A. Damasio H. Tranel D. (2013). Persistence of feelings and sentience after bilateral damage of the insula. Cereb. Cortex 23, 833–846. 10.1093/cercor/bhs077 22473895 PMC3657385

[B14] Damasio A. R. Grabowski T. J. Bechara A. Damasio H. Ponto L. L. Parvizi J.. (2000). Subcortical and cortical brain activity during the feeling of self-generated emotions. Nat. Neurosci. 3, 1049–1056. 10.1038/79871 11017179

[B15] Edgington E. S. (1995). Randomization Tests, 3rd Edn. New York, NY: Dekker.

[B16] Farb N. Daubenmier J. Price C. J. Gard T. Kerr C. Dunn B. D.. (2015). Interoception, contemplative practice, and health. Front. Psychol. 6:763. 10.3389/fpsyg.2015.00763 26106345 PMC4460802

[B17] Feinstein J. S. Khalsa S. S. Salomons T. V. Prkachin K. M. Frey-Law L. A. Lee J. E.. (2015). Preserved emotional awareness of pain in a patient with extensive bilateral damage to the insula, anterior cingulate, and amygdala. Brain Struct. Funct. 221, 1499–1511. 10.1007/s00429-014-0986-3 25577137 PMC4734900

[B18] Friman O. Borga M. Lundberg P. Knutsson H. (2003). Adaptive analysis of fMRI data. Neuroimage 19, 837–845. 10.1016/S1053-8119(03)00077-6 12880812

[B19] Fusar-Poli P. Bhattacharyya S. Allen P. Crippa J. A. Borgwardt S. Martin-Santos R.. (2010). Effect of image analysis software on neurofunctional activation during processing of emotional human faces. J. Clin. Neurosci. 17, 311–314. 10.1016/j.jocn.2009.06.027 20079652

[B20] Kelly D. H. Walter C. J. (1968). The relationship between clinical diagnosis and anxiety, assessed by forearm blood flow and other measurements. Br. J. Psychiatry 114, 611–626. 10.1192/bjp.114.510.611 5654135

[B21] Kida I. Smith A. J. Blumenfeld H. Behar K. L. Hyder F. (2006). Lamotrigine suppresses neurophysiological responses to somatosensory stimulation in the rodent. Neuroimage 29, 216–224. 10.1016/j.neuroimage.2005.07.015 16112588

[B22] Lader M. H. Wing L. (1966). Physiological Measures, Sedative Drugs, and Morbid Anxiety. Maudsley Monographs no. 14. (London, UK: Oxford University Press), 10:314 10.1016/0022-3999(66)90124-3

[B23] Mataix-Cols D. An S. K. Lawrence N. S. Caseras X. Speckens A. Giampietro V.. (2008). Individual differences in disgust sensitivity modulate neural responses to aversive/disgusting stimuli. Eur. J. Neurosci. 27, 3050–3058. 10.1111/j.1460-9568.2008.06311.x 18588543

[B24] Medford N. (2012). Emotion and the unreal self: depersonalization disorder and de-affectualization. Emot. Rev. 4, 139–144. 10.1177/1754073911430135

[B25] Medford N. Baker D. Hunter E. Sierra M. Lawrence E. Phillips M. L.. (2003). Chronic depersonalization following illicit drug use: a controlled analysis of 40 cases. Addiction 98, 1731–1736. 10.1111/j.1360-0443.2003.00548.x 14651505

[B26] Medford N. Brierley B. Brammer M. Bullmore E. T. David A. S. Phillips M. L. (2006). Emotional memory in depersonalization disorder: a functional MRI study. Psychiatry Res. 148, 93–102. 10.1016/j.pscychresns.2006.05.007 17085021

[B27] Medford N. Critchley H. D. (2010). Conjoint activity of anterior insular and anterior cingulate cortex: awareness and response. Brain Struct. Funct. 214, 535–549. 10.1007/s00429-010-0265-x 20512367 PMC2886906

[B28] Medford N. Sierra M. Baker D. David A.S. (2005). Understanding and treating depersonalisation disorder. Adv. Psychiatr. Treat. 11, 92–100. 10.1192/apt.11.2.92

[B29] Meriau K. Wartenburger I. Kazzer P. Prehn K. Villringer A. van der Meer E.. (2009). Insular activity during passive viewing of aversive stimuli reflects individual differences in state negative affect. Brain Cogn. 69, 73–80. 10.1016/j.bandc.2008.05.006 18632198

[B30] Michal M. Reuchlein B. Adler J. Reiner I. Beutel M. E. Vogele C.. (2014). Striking discrepancy of anomalous body experiences with normal interoceptive accuracy in depersonalization-derealization disorder. PLoS ONE 9:e89823. 10.1371/journal.pone.0089823 24587061 PMC3937420

[B31] Morris J. S. Friston K. J. Dolan R. J. (1998) Experience-dependent modulation of tonotopic neural responses in human auditory cortex. Proc. Biol. Sci. 265, 649–657. 10.1098/rspb.1998.0343 9608726 PMC1689028

[B32] Nelson H. E. (1982). National Adult Reading Test (NART): For the Assessment of Premorbid Intelligence in Patients with Dementia: Test Manual. Windsor, UK: NFER-Nelson.

[B33] Ochsner K. N. Gross J. J. (2005). The cognitive control of emotion. Trends Cogn. Sci. 9, 242–249. 10.1016/j.tics.2005.03.010 15866151

[B34] Ogawa S. Lee T. M. Kay A. R. Tank D. W. (1990). Brain magnetic-resonance-imaging with contrast dependent on blood oxygenation. Proc. Natl. Acad. Sci. U.S.A. 87, 9868–9872. 10.1073/pnas.87.24.9868 2124706 PMC55275

[B35] Ohira H. Nomura M. Ichikawa N. Isowa T. Iidaka T. Sato A.. (2006). Association of neural and physiological responses during voluntary emotion suppression. Neuroimage 29, 721–733. 10.1016/j.neuroimage.2005.08.047 16249100

[B36] Philippi C. L. Feinstein J. S. Khalsa S. S. Damasio A. Tranel D. Landini G.. (2012). Preserved self-awareness following extensive bilateral brain damage to the insula, anterior cingulate, and medial prefrontal cortices. PLoS ONE 7:e38413. 10.1371/journal.pone.0038413 22927899 PMC3425501

[B37] Phillips M. L. Medford N. Senior C. Bullmore E. T. Suckling J. Brammer M. J.. (2001). Depersonalization disorder: thinking without feeling. Psychiatry Res. 108, 145–160. 10.1016/S0925-4927(01)00119-6 11756013

[B38] Phillips M. L. Young A. W. Senior C. Brammer M. Andrew C. Calder A. J.. (1997). A specific neural substrate for perceiving facial expressions of disgust. Nature 389, 495–498. 10.1038/39051 9333238

[B39] Schulz A. Köster S. Beutel M. E. Schachinger H. Vögele C. Rost S.. (2015). Altered patterns of heartbeat-evoked potentials in depersonalization/derealization disorder: neurophysiological evidence for impaired cortical representation of bodily signals. Psychosom. Med. 77, 506–516. 10.1097/PSY.0000000000000195 25984819

[B40] Shcherbinin S. Doyle O. Zelaya F. O. de Simoni S. Mehta M. A. Schwarz A. J. (2015). Modulatory effects of ketamine, risperidone and lamotrigine on resting brain perfusion in healthy human subjects. Psychopharmacology (Berl). 232, 4191–4204 10.1007/s00213-015-4021-z 26223493

[B41] Sierra M. Baker D. Medford N. David A. S. (2005). Unpacking the depersonalization syndrome: an exploratory factor analysis on the Cambridge Depersonalization Scale. Psychol. Med. 35, 1523–1532. 10.1017/S0033291705005325 16164776

[B42] Sierra M. Baker D. Medford N. Lawrence E. Patel M. Phillips M. L.. (2006). Lamotrigine as an add-on treatment for depersonalization disorder: a retrospective study of 32 cases. Clin. Neuropharmacol. 29, 253–258. 10.1097/01.WNF.0000228368.17970.DA 16960469

[B43] Sierra M. Berrios G. E. (1998) Depersonalization: neurobiological perspectives. Biol. Psychiatry, 44, 898–908. 9807645 10.1016/s0006-3223(98)00015-8

[B44] Sierra M. Berrios G. E. (2000). The Cambridge Depersonalization Scale: a new instrument for the measurement of depersonalization. Psychiatry Res. 93, 153–164. 10725532 10.1016/s0165-1781(00)00100-1

[B45] Sierra M. David A. S. (2011). Depersonalization: a selective impairment of self-awareness. Conscious. Cogn. 20, 99–108. 10.1016/j.concog.2010.10.018 21087873

[B46] Sierra M. Medford N. Wyatt G. David A. S. (2012). Depersonalization disorder and anxiety: a special relationship? Psychiatry Res. 197, 123–127. 10.1016/j.psychres.2011.12.017 22414660

[B47] Sierra M. Phillips M. L. Lambert M. V. Senior C. David A. S. Krystal J. H. (2001). Lamotrigine in the treatment of depersonalization disorder. J. Clin. Psychiatry 62, 826–827. 10.4088/JCP.v62n1012b 11816874

[B48] Sierra M. Senior C. Dalton J. McDonough M. Bond A. Phillips M. L.. (2002). Autonomic response in depersonalization disorder. Arch. Gen. Psychiatry 59, 833–838. 10.1001/archpsyc.59.9.833 12215083

[B49] Simeon D. Guralnik O. Schmeidler J. Knutelska M. (2004). Fluoxetine therapy in depersonalisation disorder: randomised controlled trial. Br. J. Psychiatry 185, 31–36. 10.1192/bjp.185.1.31 15231553

[B50] Simeon D. Kozin D. S. Segal K. Lerch B. DuJour R. Giesbrecht T. (2008). De-constructing depersonalization: further evidence for symptom clusters. Psychiatry Res. 157, 303–306. 10.1016/j.psychres.2007.07.007 17959254

[B51] Simmons A. Moore E. Williams S. C. R. (1999). Quality control for functional magnetic resonance imaging using automated data analysis and Shewhart charting. Magn. Reson. Med. 41, 1274–1278. 10371463 10.1002/(sici)1522-2594(199906)41:6<1274::aid-mrm27>3.0.co;2-1

[B52] Spielberger C. (1983). Manual for the State-Trait Anxiety Inventory (STAI). PaloAlto, CA: Consulting Psychologists Press.

[B53] Tabbert K. Stark R. Kirsch P. vaitl D. (2005). Hemodynamic responses of the amygdala, the orbitofrontal cortex and the visual cortex during a fear conditioning paradigm. Int. J. Psychophysiol. 57, 15–23. 10.1016/j.ijpsycho.2005.01.007 15935259

[B54] Talairach J. Tournoux P. (1988). Co-planar Stereotactic Atlas of the Human Brain. Stuttgart: Thieme.

